# Efficacy comparison of vascularized iliac crest bone flap and Ilizarov bone transport in the treatment of traumatic bone defects of the tibia combined with large soft tissue defects

**DOI:** 10.1186/s13018-023-03783-9

**Published:** 2023-05-11

**Authors:** Zhe-ming Cao, Xin-lei Sui, Yu Xiao, Li-ming Qing, Pan-feng Wu, Ju-yu Tang

**Affiliations:** Department of Orthopedics, Xiangya Hospital, Central South University, 87 Xiangya Road, Changsha, 410008 Hunan China

**Keywords:** Tibia defects, Vascularized iliac crest bone flap, Ilizarov bone transport, Comparative study

## Abstract

**Background:**

Traumatic tibial defect complicated with soft tissue defect is a difficult problem in clinic. Vascularized iliac crest bone flap (VIBF) and Ilizarov bone transport are effective methods to treat tibial defects with limited defect length, which most need to be explored accordingly.

**Methods:**

In this study, a total of 68 patients with traumatic tibial defect (ranging from 4 to 10 cm) and large soft tissue defect were collected retrospectively. The soft tissue defects were repaired by latissimus dorsal musculocutaneous flap (LD), anterolateral thigh flap (ALTF) or both. Thirty-three cases were treated with vascularized iliac crest bone flap transplantation and 35 cases were treated with Ilizarov bone transport. Intraoperative and postoperative follow-up data (including operation time, blood loss, bone union time, external fixation time, external fixation index, complication rate, reoperation rate, and functional evaluation) were recorded, and comparative analysis was performed.

**Results:**

The median follow-up time was 32 months. Compared with Ilizarov group, the VIBF group exhibited statistically faster bone union time (6.3 ± 1.0 vs. 18.2 ± 3.0 months). Moreover, the VIBF group showed shorter EFT (7.3 ± 1.0 vs. 19.2 ± 3.0 months) and a better EFI (34.8 ± 9.2 vs. 84.2 ± 23.7 days/cm). The excellent and good rate of lower limb appearance evaluation in VIBP group was significantly better than that in Ilizarov group. The complication rate and reoperation rate were significantly higher in Ilizarov group.

**Conclusion:**

In summary, compared with Ilizarov bone transport, VIBP has the advantages of faster healing, shorter external fixation time, lower complication and reoperation rate, and better appearance within the limited defect length. Ilizarov bone transport is still preferred when the defect length exceeds the maximum repair length of the iliac flap. The daily handling required by bone transport process is painful.

**Level of evidence:**

III, Case–control study.

## Introduction

Traumatic tibial defects combined with soft tissue defects are very common in clinical practice. Open tibia fractures often lead to bone infection, osteonecrosis and chronic osteomyelitis without prompt treatment due to the lack of blood supply and insufficient local soft tissue. It causes long treatment period, high spending along with damaging effects on appearance and function of lower limbs [1–3]. Removal of the dead bone inevitably leads to a tibial defect which poses a difficult problem for the reconstructive surgeons.

Currently, there are multiple approaches to repair segmental tibia defects including autogenous or allogeneic bone graft [4–6], nonvascular fibula graft [7, 8], vascularized fibular or iliac crest bone flap transplantation [9–14], Ilizarov bone transport [15–19], Masquelet technique [20–23], and so forth. All of them presented good efficacy in repairing tibial defects. Usually, the surgeon will select the proper method according to the patient condition and their personal experience.

Vascularized iliac crest bone flap transplantation (VIBF group for short) and Ilizarov bone transport (Ilizarov group for short) are both effective methods for tibia defects. Owing to abundant blood supply, vascularized iliac crest bone flap promotes bone healing and has a strong ability against infection. While Ilizarov bone transport is a main treatment for bone defects with simple operation and less surgical trauma.

In this study, we compared vascularized iliac crest bone flap transplantation and Ilizarov bone transport for the reconstruction of traumatic tibia defects combined with large soft tissue defects. We evaluated the outcome, complication, reoperation, appearance and function.

## Material and methods

### Patients

This retrospective study enrolled 68 cases (37 males and 31 females) of traumatic tibia defects combined with large soft tissue defects admitted to our hospital over the years 2014 to 2020. The cause of injury included vehicle accident in 36 cases, fall from height in 5 cases, bruise injury in 12 cases and machine injury in 15 cases. 33 cases were treated with vascularized iliac crest bone flap transplantation and 35 cases were treated with Ilizarov bone transport. The same surgical team performed all the procedures.

The inclusion criteria are as follows: (1) the tibial and soft tissue defects caused by trauma and older than 18 years of age. (2) After debridement, tibial defects ranged from 4 to 10 cm, and diaphyseal defects. (3) The tibia defect reconstructed by vascularized iliac crest bone flap transplantation or Ilizarov bone transport. (4) The soft tissue defects repaired by latissimus dorsal musculocutaneous flap (LD), anterolateral thigh flap (ALTF), or both. (5) No underlying diseases or serious comorbidities. (6) The follow-up time was longer than 2 years.

The exclusion criteria are listed below: (1) the tibia defect with the symptom of adjacent joint infection. (2) Patients with metabolic bone diseases and bone tumors. (3) Old patients (> 65 years) or patients with vascular diseases. (4) Patients with severe cardiopulmonary dysfunction, liver and kidney dysfunction and other systemic diseases. (5) Patients who were lost to follow-up.

### Surgical technique

Preoperative preparation includes laboratory and imaging examinations. CT angiography (CTA) and color ultrasound were carried out to check the continuity of the main vessels, vascular diseases or anatomical variation.

All the devitalized tissues including dead bone, infected or necrotic tissues were removed by radical debridement. Afterwards, deep tissue samples were collected for bacterial identification, susceptibility study and histopathological examination. The length of tibia defects was measured.

**Fig. 1:**
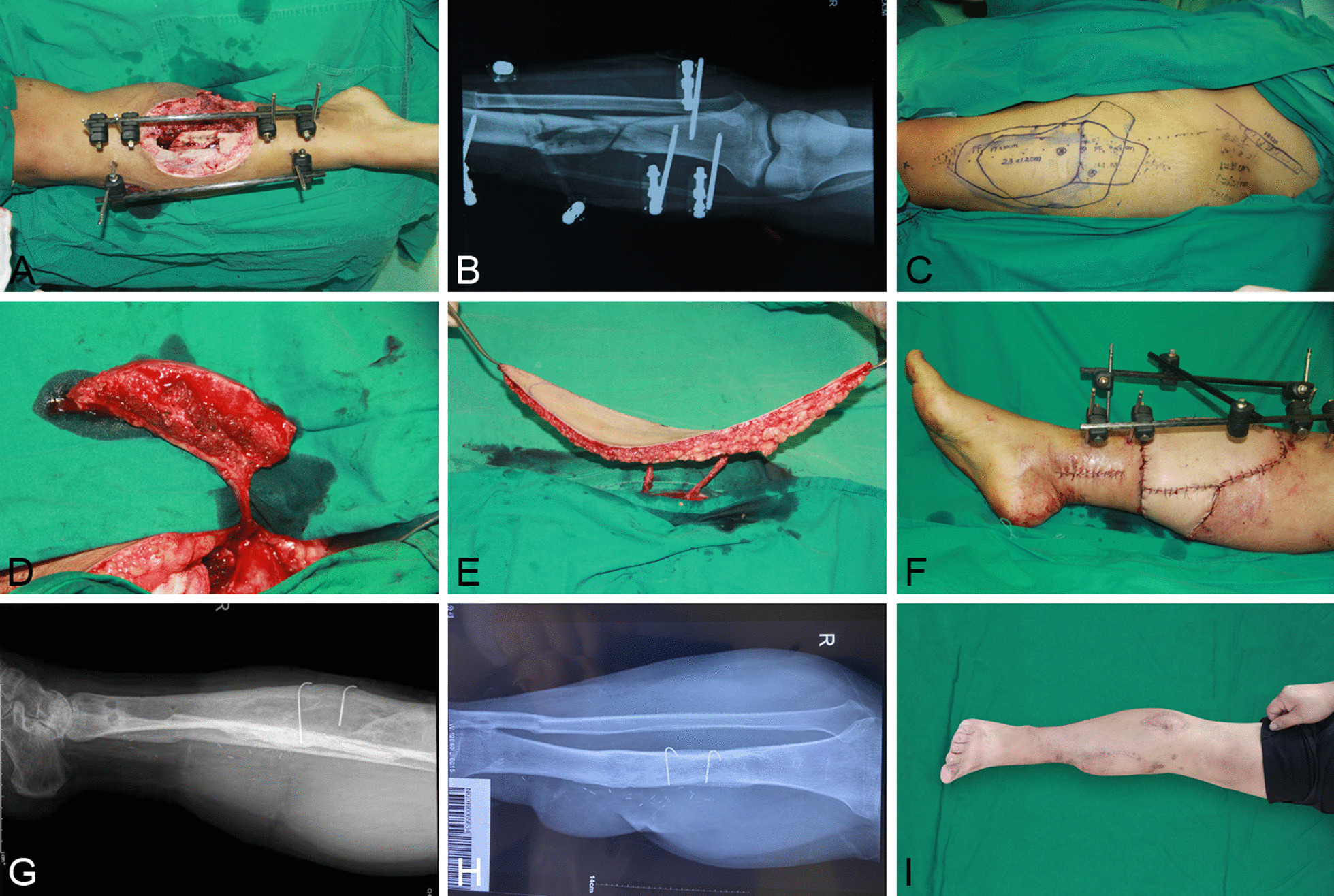
A 29-year-old male presented with an open tibia and fibula fracture due to traffic injury. **A** Tibia and soft tissue defects after debridement. **B** Preoperative X-ray. **C** The designed ALTP and iliac flap of donor site. **D**, **E** Free ALTF and iliac flap was harvested. **F** Postoperative view of ALTF and iliac flap transplantation. **G** Postoperative follow-up was 8 months, and X-ray films were taken 2 month after removal of the external fixator. **H**, **I** Postoperative view and X-ray of the affected limb 24 months after surgery

Patients treated with vascularized iliac crest bone flap transplantation underwent debridement, external fixation, iliac crest bone flap and skin flap (LD, ALTF, or both) dissection and transplantation, Kirschner wires fixation in docking sites and vascular anastomosis. Notably, we anastomosed recipient vessels (anterior or posterior tibial vessels) to the proximal side of the skin flap T-shaped vascular pedicle (descending branch of lateral circumflex femoral vessels or thoracodorsal vessels) and the distal side to the deep iliac circumflex vessels (Fig. 1).

Patients treated with Ilizarov bone transport experienced two-stage operation. The primary operation included debridement, external fixation, free flap transplantation for soft tissue reconstruction. After the skin flap survived stably, approximately 6 weeks later, second-stage surgery was performed to install Ilizarov fixator. Postoperatively, anticonvulsants, anticoagulants and sensitive antibiotics were used. Early functional exercise was begun on the second postoperative day.

**Fig. 2:**
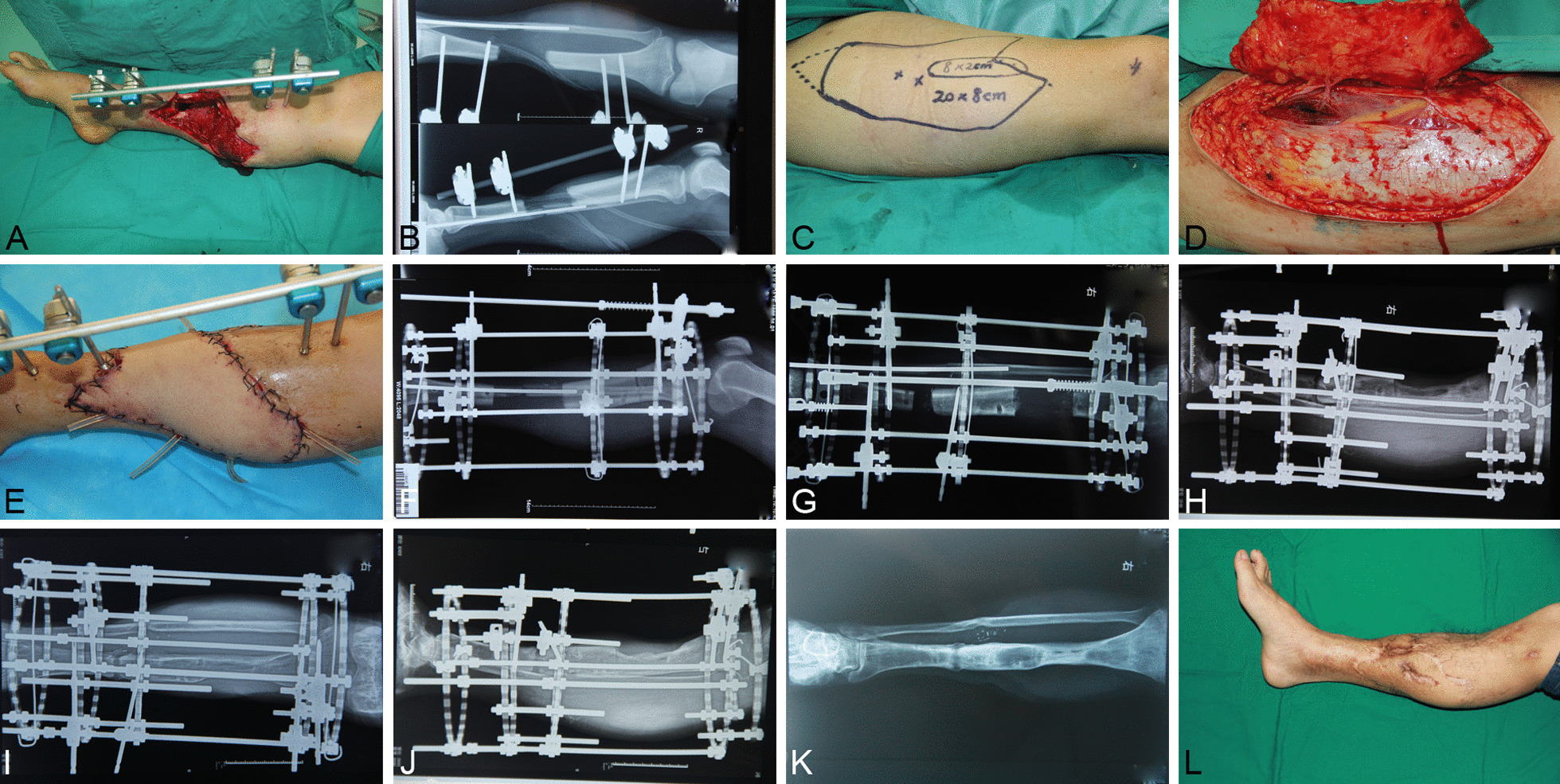
A 26-year-old male presented with an open tibia and fibula fracture due to traffic injury. **A** The right leg after radical debridement. **B** Preoperative X-ray, **C** The designed ALTP flap of donor site. **D** Free ALTF was harvested. **E** Postoperative view of ALTF transplantation. **F** X-ray after Ilizarov bone transport. **G**–**J** Postoperative radiographic view at the 3-month, 12-month, 18-month, and 24-month follow up. **K** Postoperative follow-up was 30 months, and X-ray films were taken 1 month after removal of the external fixator. **L** Postoperative view of the affected limb 30 months after surgery

In Ilizarov group, bone transport was started after 5–7 days latent period at a rate of 1 mm per day. Partial weight bearing began at 2 weeks. The Ilizarov fixators were removed when complete bone healing at the docking site was achieved radiographically and clinically (Fig. 2).

In VIBF group, patients were allowed to a partial weight bearing with double crutches at 1 month after surgery and encouraged to gradually bear more weight until they could walk with full weight bearing. External fixators were removed after bone union confirmed by X-ray.

### Follow-up evaluation

Operation duration and blood loss were recorded. Follow-up was performed at 1, 3, 6, 9, and 12 months postoperatively and every 6 months after bone union. Bone healing time, external fixation time (EFT), external fixation index (EFI), complications and reoperations were recorded. Bone and functional results were evaluated by the Association of the Study and Application of the Method of Ilizarov (ASAMI) [24, 25]. The appearance was evaluated subjectively and objectively [26].

### Statistic analysis

Statistical analysis was performed by SPSS 20.0 software (SPSS Inc., US). Quantitative data were expressed as means ± standards (standard deviation) and compared using the student’s *t*-test. Qualitative data were expressed as numbers or percentages and compared using the *χ*^2^ test and Fisher's exact test. *p* < 0.05 was considered statistically significant.

## Results

The average follow-up was 32 months (range 24–40). Tibia defects and soft tissue defects were repaired in all patients.

A total of 68 patients with tibia defects accompanied by soft tissue defects were included. Among these 33 cases underwent VIBP and 35 cases with Ilizarov bone transport. After debridement, the average length of tibial defect in both groups was 6.7 cm (range 4–10 cm). The soft tissue defect size was 166.7 ± 41.0 cm^2^ in VIBF group and 164.9 ± 32.0 cm^2^ in Ilizarov group. The demographic data and clinical characteristics of 68 patients were shown in Table 1, and there was no statistical difference between the two groups. The homogeneity of the two groups was good, and the results of short- and long-term follow-up could be considered comparable.

**Table 1:** Demographic data

Variable	VIBF group (*N* = 33)	Ilizarov group (*N* = 35)	*p* value^#^
Age (year)	32.3 ± 10.7	33.2 ± 11.9	0.738
Gender			0.635
Male	19	18	
Female	14	17	
Smoking History			0.626
No	17	21	
Yes	16	14	
BMI			0.836
< 25 kg/m^2^	20	19	
≥ 25–29.9 kg/m^2^	8	9	
≥ 30 kg/m^2^	5	7	
Tibia defect site			0.808
Middle	16	15	
Lower segment	17	20	
Bone defect length (cm)	6.7 ± 1.7	6.7 ± 1.7	0.885
Soft tissue defect size (cm^2^)	166.7 ± 41.0	164.9 ± 32.0	0.834
Soft tissue repair			0.963
ALTP	17	17	
LD	11	12	
Other*	5	6	
Bacterial culture			0.995
*Staphylococcus aureus*	10	11	
*Pseudomonas aeruginosa*	8	9	
*Escherichia coli*	7	6	
MRSA	3	3	
None	5	6	

*VIBF* vascularized iliac bone flap, *BMI* body mass index, *ALTP* anterolateral thigh perforator, *MLD* modified latissimus dorsi musculocutaneous, *MRSA* Methicillin-resistant *Staphylococcus aureus*

*Combination to repair the wound

^#^Two-sided Fisher’s exact test or Student’s t-test

The flap size was 181.5 ± 40.9 cm^2^ in VIBF group and 180.3 ± 33.0 cm^2^ in Ilizarov group. No statistical differences were found between the two groups in flap harvest time and external fixator installation time. The VIBF group took an additional 41.7 ± 8.4 min to harvest the iliac crest bone flap. The blood loss in VIBF group was statistically more than Ilizarov group (315.2 ± 56.6 vs. 218.6 ± 34.5 ml, *p* < 0.05). Total blood loss in VIBF group was statistically more than Ilizarov group (315.2 ± 56.6 vs. 259.1 ± 33.8 ml, *p* < 0.05) (Table 2).

**Table 2:** Intraoperative data, short-term and long-term follow-up results of soft tissue repair surgery

Variable	VIBF group (*N* = 33)	Ilizarov group (*N* = 35)	*p* value
Flap size (cm^2^)	181.5 ± 40.9	180.3 ± 33.0	0.893
Flap harvested time (min)	47.5 ± 8.3	48.3 ± 9.7	0.746
Bone flap harvested time (min)	41.7 ± 8.4	–	–
External fixation installation time (min)	26.7 ± 4.4	26.4 ± 4.5	0.826
Operative blood loss(ml)	315.2 ± 56.6	218.6 ± 34.5	0.001
Flap complications			1.000
Total flap necrosis	0	0	
Partial flap necrosis	2	3	
Factors of flap necrosis			–
Vascular crisis	0	0	
Infection	1	2	
Hematoma	1	1	
Donor site morbidity			0.608
Delayed wound healing	2	1	
Cosmetic evaluation			
Subjectively^a^	26/7	17/18	0.013
Excellent	17	12	
Good	9	5	
Moderate	6	10	
Poor	1	8	
Objectively^b^	27/6	19/16	0.020
Excellent	18	13	
Good	9	6	
Moderate	6	9	
Poor	0	7	

^a^Guardians of the patients

^b^Blinded third-party observer

Regarding flap-related complications, partial necrosis occurred in 2 patients in the VIBF group (including 1 infection and 1 hematoma) and in 3 patients in the Ilizarov group (including 2 infection and 1 hematoma). Delayed wound healing in donor site took place in 2 patients of VIBF group and 1 patient of Ilizarov group. The two groups of patients achieved wound healing after careful dressing. The excellent and good rates of the overall lower extremity appearance evaluation from patients and blinded third-party observer in the Ilizarov group were 48.6% and 54.3% respectively, which were significantly lower than the excellent and good rates of 78.8% and 81.8% in the VIBP group (*p* < 0.05) (Table 2).

**Fig. 3:**
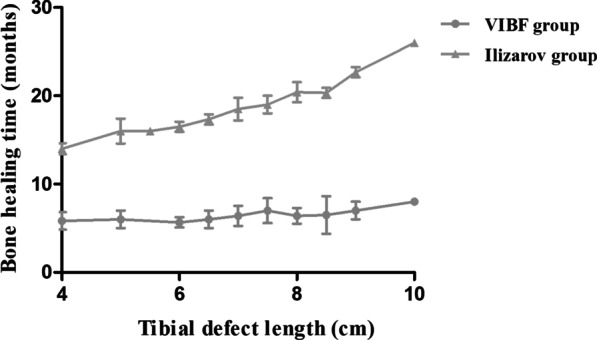
The effect of VIBF and Ilizarov group on the change trend of bone healing time under different tibial defect length

The average second-stage Ilizarov fixator installation time was 56.7 min. Compared with Ilizarov group, the VIBF group exhibited statistically faster bone union time (6.3 ± 1.0 vs. 18.2 ± 3.0 months, *p* < 0.05). The comparison of tibia healing time under different bone defect length showed that VIBF group was significantly better than Ilizarov group (*p* < 0.05, Fig. 3). Moreover, the VIBF group showed shorter EFT (7.3 ± 1.0 vs. 19.2 ± 3.0 months, *p* < 0.05) and a better EFI (34.8 ± 9.2 vs. 84.2 ± 23.7 days/cm, *p* < 0.05) (Table 3).

**Table 3:** Intraoperative data, short-term and long-term follow-up results of bone defect repair

Variable	VIBF group (*N* = 33)	Ilizarov group (*N* = 35)	*p* value^#^
Ilizarov-external fixation installation time (min)	–	56.7 ± 5.4	–
Operative blood loss (ml)	–	40.6 ± 8.0	–
Total blood loss (ml)	315.2 ± 56.6	259.1 ± 33.8	0.001
External fixed carrying time (months)	7.3 ± 1.0	19.2 ± 3.0	0.001
External fixation index	34.8 ± 9.2	84.2 ± 23.7	0.001
Bone healing time (months)	6.3 ± 1.0	18.2 ± 3.0	0.001
Reoperation	0	7	0.011
Complication	2	10	0.024
Deep pin tract infection	2	3	
Pin loosening	0	1	
Soft tissue incarceration	0	3	
Delayed or non-union fracture	0	3^&^	
Refracture	0	0	
ASAMI bone results	31/2	32/3	1.000
Excellent	21	19	
Good	10	13	
Fair	2	3	
Poor	0	0	
ASAMI functional results	31/2	30/5	0.674
Excellent	20	19	
Good	11	11	
Fair	2	5	
Poor	0	0	

^&^Delayed or non-union fracture was treated with iliac cancellous bone graft

^#^indicates reoperation statistical results of the two groups were (*P* = 0.011), and complication rate was (*P* = 0.024), with *P* values less than 0.05, showing statistical differences

During the follow-up period, there was 2 cases of pin tract infection in the VIBF group. In the Ilizarov group, there were 3 cases of pin tract infection, 1 case of pin loosening, 3 cases of soft tissue incarceration, and 3 cases of delayed or non-union fracture. The pin tract infection was well controlled with antibiotic treatment and dressing. 7 cases complications in the Ilizarov group required further revision surgery. The complication rate and reoperation rate were significantly higher in Ilizarov group (*p* < 0.05, Table 3). In addition, the excellent and good rates of bone and functional outcomes of ASAMI in the VIBF group were 93.9% and 93.9%, respectively. While in Ilizarov group, the scores were 91.4% and 85.7% respectively. There was no statistical difference between the two groups.

## Discussion

Large tibial bone defect combined with large soft tissue defect is a thorny problem in clinic. As the first step is to solve the problem of wound repair, the condition of the wound base of the defect is more serious than that of the common soft tissue defect (the wound is large and the infection is serious). As a result, the risk of necrosis after skin flap coverage increases, as well as long-term postoperative wound non-union and effusion, requiring long-term antibiotic treatment to control the corresponding situation [27]. For soft tissue repair, we prefer to use LD or ALTF, rather than local flap, to cover the soft tissue defects in the leg. There are two reasons for this. Firstly, in most open injuries, local soft tissue conditions of the lower leg have been compromised which may increase the risk of infection and flap crisis. Secondly, the donor area on the back and thighs is more concealed than the lower leg. Here we emphasize the importance of soft tissue repair. It is essential to provide a healthy soft tissue coverage for tibial healing in the management of infectious tibial defects [28–30].

However, bone defect repair is also a thorny problem, and different repair methods are limited by the length of the defect. Relevant studies have shown that each has its own advantages and disadvantages, but which effect is better is not determined. Sufficient autologous cancellous bone is usually not available in longer tibia defects [4, 5]. Allogenic bone graft is often associated with immune rejection problems, leading to infection, allogeneic bone resorption and failure of reconstruction [6]. Without enough blood supply, nonvascularized fibula flap takes longer bone union time and often accompanied by different degrees of bone resorption [7, 8]. In cases of large tibia defects, Masquelet technique also faces the problem of limited bone volume during the second stage of Masquelet procedure [20, 21]. In this study, patients with a 4–10 cm defect range were included, which is also clinically common and suitable for vascularized iliac bone flap and Ilizarov bone graft in the repair. Therefore, the purpose of this study was to explore the optimal operation of the two techniques in this range.

In 1978, Taylor et al. [31] reported the anatomical basis of vascular iliac crest bone flap through ink perfusion, and applied the bone flap to repair tibia defects in 2 patients. Subsequently, the vascular iliac crest bone flap was typically used for reconstruction of the mandible and tibia [12, 32–34]. Compared with fibular flap, which is more commonly used to repair bone defects, we chose the iliac crest bone flap because it has both cortical and cancellous bone. Cortical bone provides support, and cancellous bone provides a better microenvironment for osteogenesis, which helps to better osteogenesis and faster bone union than fibular flap [13]. In addition, the iliac crest bone flap has a rich blood supply and is more resistant to infection. Green et al. [35] suggested that vascularized fibula grafts are more suitable for skeletally immature patients who have greater potential to remodel, and it takes long time for fibular hypertrophy to bear weight. In our VIBF group of 33 cases, the tibia healed within 5–8 months after the operation. The weight-bearing walking recovered normally with no infection recurrence or refracture occurred. The appearance and function were satisfactory. Similar to our results, Zheng et al. [14] repaired 5 cases of soft tissue and bone defects with deep iliac circumflex osteocutaneous flap among which 4 cases were tibial defects. The length of the harvested iliac crest bone flap was between 6.4 and 10.5 cm with an average bone union time of 5.5 months (range 4–7 months). All the 5 patients recovered their ability to walk with only 1 patient had necrosis of the skin flap. No other complications occurred.

In particular, the maximum length of the iliac crest bone flap from Zheng et al. [14] was 10.5 cm, while it was 10 cm in our study. Owing to the irregular shape of the iliac crest, the more backward the bone flap we harvested, the more curved and the thinner the bone flap becomes. In addition, the maximum length of the iliac bone flap available was closely connected with height, weight and pelvic size. Further epidemiological investigations with large samples are needed for confirmation. In contrast, the Ilizarov technique doesn’t have the limitation on lengths. Besides, VIBF procedure requires skillful microsurgical technique which makes it more challenging operationally.

In the nineteenth century, Ilizarov summarized the stretch-stress rule and creatively invented the Ilizarov ring external fixator [36]. Since then, Ilizarov technique has been extensively used in limb extension and orthopedics. Sigmund et al. [16] treated 27 cases of tibial defects with Ilizarov bone transport. The mean bone defect size measured 5.9 cm (range 3–10), and the mean frame time was 10.3 months (range 7–17). EFI was 1.8 months/cm (range 0.9–2.7). The excellent and good rate of ASAMI bone score and function score were 100% and 92% respectively. The reoperation rate was 56% during external fixation period and 28% after removal of external fixation. Aktuglu et al. [37] retrospectively analyzed 24 patients with a mean tibial defect of 7 cm (range 5–18). The EFT was 275.5 days (range 190–437) and EFI was 52 days/cm (range 34.8–62.8). Chai et al. [19] reported a combination use of the neurocutaneous flap and the Ilizarov technique in managing trauma-related severe composite soft tissue and bone defects in the tibia. The mean distraction length and duration of use of the external fixator were 6 cm (range 4–9) and 11.4 months (range 7–20), respectively. Five patients had superficial pin-track infections and 4 patients developed mild contracture of the Achilles tendon.

In the study, we adopted Ilizarov bone transport for 35 cases of tibial defects. The mean defect length and bone union time was 6.7 cm (range 4–10) and 18.2 months (range 13–26), respectively. The EFT and EFI were 19.2 months and 84.2 days/cm. The reoperation rate was 20.0%. ASAMI bone score and function score were 91.4% and 85.7% in excellent and good rate. Compared with other centers mentioned above, our cases showed a longer bone union time and external fixation time. The reasons may be as follows. First, most of the patients suffered severe trauma accompanied by large soft tissue defects in the leg and poor general condition like anemia and hypoproteinemia. Second, some patients cannot tolerate the daily adjustment of the Ilizarov fixator due to severe pain. These patients failed to adjust on time resulting in a prolonged course of the disease.

However, Ilizarov bone transport has a high complication and reoperation rate. Pin tract infection is the most frequent complication. Others include soft tissue incarceration, union on docking site, poor mineralization of extending bone, migration of force line, ankylosis, refracture and so on. The number of complications in the Ilizarov group was 28.6% and the rate of reoperation was 12.5%. It has been reported that the average number of complications per patient range from 0.67 to 2.27 [15]. The average number of complications reported by Aktuglu et al. [37] was just 0.29 and the reoperation rate was 12.5%. In the study of Sigmund et al. [16] reoperation rate during Ilizarov external fixation was 56% (15/27) and 37% (10/27) after removal of the external fixator. As declared by Chai et al. [19] the complication rate was 50%, and there was no case of reoperation.

In this study, the advantages and disadvantages of the two groups were found as follows: (1) In the Ilizarov group, bone tissue repair could not be performed until the basic condition of the patients was adjusted and the skin flap healed completely (about 2 months). The iliac crest group is performed simultaneously with a flap and bone flap graft, thus reducing the pain of a second operation. (2) The healing time of Ilizarov group was significantly longer than that of iliac flap group, and the incidence of complications was higher. Therefore, it is inevitable that multiple surgeries are needed to solve the corresponding problems. Most patients cannot tolerate the pain brought by the treatment process and choose to remove Ilizarov external fixation, which undoubtedly increases the economic burden of patients. Iliac bone flap group can effectively solve the problem of wound coverage and bone defect in one stage, and has the advantages of short healing time, low complication rate and low cost. (3) In the Ilizarov group, the traction process may lead to scars and bloated appearance of lower limb hyperplasia, which may require further surgery for corresponding management. Therefore, iliac bone flap combined with ALTP or LD can be preferred for tibial defect with limited length. Related studies have shown that deep circumflex iliac chimeric perforator flap can repair soft tissue defect and bone defect simultaneously. However, the perforator flap has a limited resection area and is preferred when the soft tissue defect area is small [38, 39]. All patients included in this study had large soft tissue defects. Therefore, iliac bone flap combined with ALTP or LD can be preferred for tibial defect with limited length. Ilizarov bone transport is still preferred when the defect length exceeds the maximum repair length of the iliac bone flap. Since both iliac bone flap and perforator flap techniques require relatively high microsurgical foundation, they can be gradually mastered through corresponding training. It can provide different options for bone and soft tissue defect repair.

There are some limitations in this paper. First, this study was a retrospective study, which may lead to information bias. Second, the sample size was limited. However, this study is still reasonable comparative study to evaluate the efficacy in two groups of patients with homogeneous tibial defects combined with soft tissue defects.

## Conclusion

In summary, compared with Ilizarov bone transport, the vascularized iliac crest bone flap has the advantages of faster healing, shorter external fixation time, lower complication and reoperation rate, and better appearance within the limited defect length. Ilizarov bone transport is still preferred when the defect length exceeds the maximum repair length of the iliac flap. The daily handling required by bone transport process is painful.

## Abbreviations

ALTP: Anterorlateral thigh perforator
LD: Latissimus dorsi musculocutaneous
BMI: Body mass index
ASAMI: Association for the Study and Application of the Method of Ilizarov
EFT: External fixation time
EFI: External fixation index
VIBF: Vascularized iliac crest bone flap
CTA: CT angiography
